# Obesity and Heart Failure: Mechanistic Insights and the Regulatory Role of MicroRNAs

**DOI:** 10.3390/genes16060647

**Published:** 2025-05-28

**Authors:** Parul Sahu, Furkan Bestepe, Sezan Vehbi, George F. Ghanem, Robert M. Blanton, Basak Icli

**Affiliations:** Molecular Cardiology Research Institute, Department of Medicine, Tufts Medical Center, Boston, MA 02111, USA; parul.sahu@tuftsmedicine.org (P.S.); furkan.bestepe@tuftsmedicine.org (F.B.); svakhpieva17@ku.edu.tr (S.V.); george.ghanem@tuftsmedicine.org (G.F.G.); robert.blanton@tuftsmedicine.org (R.M.B.)

**Keywords:** microRNAs, obesity, heart failure, RNA-based therapies

## Abstract

Heart failure (HF) remains a leading cause of morbidity and mortality worldwide, driven by diverse pathophysiological mechanisms. Among its major risk factors, obesity has emerged as a lobal public health concern affecting individuals across all age groups. The rising prevalence of obesity significantly increases the risk of cardiovascular complications, including the development and progression of HF. MicroRNAs (miRNAs), small non-coding RNA molecules, have garnered attention for their regulatory roles in cardiovascular disease, particularly through post-transcriptional modulation of gene expression. This review highlights the involvement of miRNAs in key pathological processes observed in the obese heart, including cardiac remodeling, apoptosis, angiogenesis, inflammation, mitochondrial dysfunction, and myocardial lipotoxicity. Understanding how specific miRNAs and their targets contribute to HF in the context of obesity may inform the development of novel RNA-based therapeutic strategies for cardiometabolic disease.

## 1. Introduction

HF is a significant global health problem, affecting over 56 million adults worldwide. Conditions such as coronary heart disease (CHD), diabetes, obesity, and smoking collectively account for 52% of HF cases [1]. Among these, accumulating evidence points to obesity, particularly elevated body mass index (BMI) and fluctuations in BMI, as a major contributor to HF by increasing cardiovascular stress, exacerbating the cardiac hemodynamic load, and promoting structural and functional changes [2,3]. These obesity-driven changes result in higher rates of cardiovascular-related mortality [4,5,6,7]. For instance, findings from the Framingham Heart Study and others have shown that a one-unit increase in BMI increases the risk of HF by 5% in men and 7% in women [8,9,10]. 

Importantly, regional adipose distribution, rather than total body adiposity, may play an essential role in the progression of cardiovascular disease (CVD) to HF. Visceral adipose depots, such as intra-abdominal (VAT) [11,12] and the epicardial adipose tissue (EAT), a unique fat depot located between the myocardium and the visceral layer of the epicardium, have been implicated in HF development [13]. However, the precise mechanisms linking obesity-associated fat depots to HF pathophysiology are not yet entirely understood.

Over the past 50 years, global obesity rates have nearly tripled, fueling a widespread epidemic [14]. The significant rise in obesity is the leading cause of the rapid expansion of diabetes mellitus (DM) [15] and contributes directly to CVD-associated morbidity and mortality worldwide [16]. Excessive accumulation of adipose tissue can lead to multifactorial cardiovascular complications, such as atherosclerosis [17], hypertension [18], and left ventricular (LV) systolic and diastolic dysfunction [19], all of which can progress into the development of heart failure (HF). 

The two main types of heart failure are heart failure with reduced ejection fraction (HFrEF) and heart failure with preserved ejection fraction (HFpEF) [20,21]. HFrEF is defined by an LVEF of less than 40% and is characterized by impaired systolic function, where the heart muscle cannot contract effectively to pump blood. In contrast, HFpEF is defined by an LVEF of 50% or greater and is primarily associated with diastolic dysfunction, in which the heart muscle maintains contractility but has impaired relaxation and filling capacity [20]. A recent study suggested that obesity and DM confer a greater risk of HFpEF compared to HFrEF, particularly in women [22]. Approximately half of individuals affected by obesity experience HFpEF, with patients who have DM exhibiting a more substantial disease burden [23]. This is evidenced by advanced cardiac imaging techniques, such as myocardial strain analysis, which reveal subclinical myocardial dysfunction in patients with DM [24].

MicroRNAs (miRNAs) are essential regulators of multiple biological processes. These small (∼20–22 nt), single-stranded, non-coding RNAs are evolutionarily conserved and regulate gene expression at the post-transcriptional level. MiRNAs are critical in maintaining cellular and systemic homeostasis, making them pivotal in understanding HF pathophysiology. They bind to target mRNAs through base complementarity with the 3′ UTR region, leading to inhibition of their translation or the promotion of mRNA degradation. More than 2000 miRNAs have been identified in human eukaryotic cells [25], with functions spanning both physiological and pathophysiological conditions. 

Accumulating studies reveal that miRNA dysregulation may impact cardiovascular disease states and they play distinct roles in both HFpEF and HFrEF [26]. For example, in patients with chronic HF, heart and muscle-specific miRNAs, known as myomiRs, positively correlate with indices of myocardial damage, including cardiac troponin levels [27]. In patients with DM and HFpEF, treatment with the sodium-glucose cotransporter 2 (SGLT2) inhibitor empagliflozin resulted in significant downregulation of miR-126, miR-342-3p, and miR-638. Conversely, miR-21 and miR-92 were significantly upregulated in the blood following empagliflozin treatment [28].

**Figure 1:**
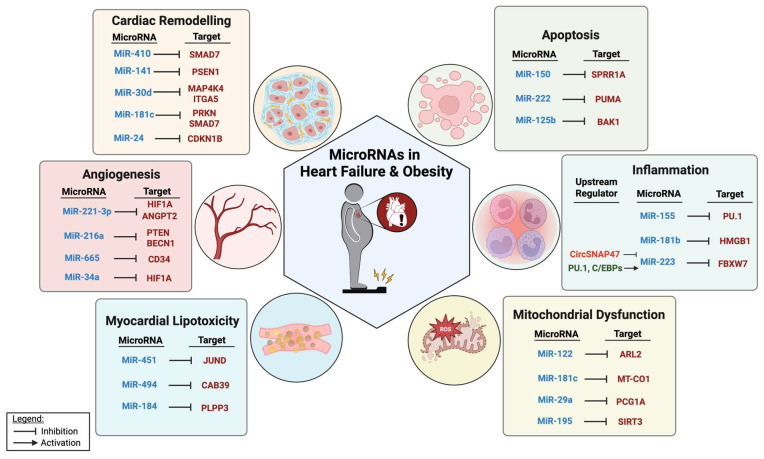
MiRNAs and their targets in pathological processes relevant to HF in the context of obesity.

Here, we provide an overview of the role of miRNAs in modulating various aspects of HF in the context of obesity, including cardiac remodeling, inflammation, angiogenesis, apoptosis, mitochondrial dysfunction, and lipotoxicity (Figure 1). Elucidating the role of miRNAs in HF offers new insights into the pathobiology underlying the increased risk of developing HF associated with DM and obesity. This, in turn, may provide novel opportunities for therapeutic intervention.

## 2. Cardiac Remodeling

Cardiac remodeling commonly refers to changes in cardiac size, shape, and function, including cardiac hypertrophy and fibrosis, in response to injury or pathological stress. Obesity is closely linked to cardiac remodeling, primarily due to the increased hemodynamic demand on the heart. This elevated demand leads to LV enlargement and dysfunction, ultimately progressing to HF. The underlying mechanisms of this progression involve pathological processes such as myocardial hypertrophy and cardiac fibrosis [29]. 

Myocardial hypertrophy refers to the abnormal enlargement of heart muscle cells in response to increased workload, while cardiac fibrosis involves the excessive deposition of extracellular matrix components, which stiffens the heart muscle and impairs its function [30]. Recent studies have highlighted the significant role of miRNAs in cardiac remodeling by regulating gene expression and cellular processes in the heart. 

Several miRNAs, such as miR-1 [31,32] miR-133a [33,34,35], miR-30d-5p [36], miR-208a [37] miR-541-5p [38], miR-217 [39], miR-155 [40], miR-199b-5p [41], and miR-378-3p [42] have been identified as key regulators of cardiac remodeling. Notably, certain miRNAs have been identified as upregulated in the plasma of obese individuals, where they show strong correlations with the molecular pathways involved in obesity-induced HF. These miRNAs likely contribute to the pathophysiology of obesity-related cardiac dysfunction by modulating key genes involved in hypertrophy and fibrosis. For example, in a rat model of diet-induced obesity (DIO)**,** miR-410-5p was significantly upregulated in cardiac tissue, where it targeted and inhibited SMAD7 expression, leading to increased SMAD2 phosphorylation and subsequent activation of fibrosis. Conversely, the neutralization of miR-410-5p in obese rats mitigated cardiac remodeling by decreasing fibrosis. Interestingly, tissue-specific overexpression of miR-410-5p in kidneys or adipose tissue resulted in increased miR-410-5p expression in the heart and in circulating exosomes, suggesting a potential systemic role of miR-410-5p in obesity-associated cardiac dysfunction [43].

MiR-141-3p and miR-144-3p were found to be significantly increased in the myocardium of obese rats, with opposing roles in obesity-associated cardiac remodeling. The inhibition of miR-141-3p or overexpression of miR-144-3p reduced palmitate-induced cardiac hypertrophy and fibrosis, while the overexpression of miR-141-3p or inhibition of miR-144-3p exacerbated cardiac dysfunction [44]. In support of these findings, miR-141-3p has been shown to target presenilin 1 (PSEN1) and inhibit the Notch1/PTEN/AKT pathway. Blocking this pathway reversed miR-141-3p's effects on palmitate-induced cardiomyocyte apoptosis [45]. Interestingly, treatment with the SGLT2 inhibitor ipragliflozin reduced both miR-144-3p expression and cardiac hypertrophy in Dahl-sensitive obese rats, suggesting potential tissue- or disease-specific effects of miR-144-3p, such as having protective effects during early remodeling but contributing to maladaptive responses in later stages or under different metabolic conditions [46]. 

MiR-30d-5p was significantly elevated in patients with HF [36] and obesity [47]. In murine models of ischemic HF, the overexpression of miR-30d-5p improved cardiac function by reducing myocardial fibrosis and attenuating apoptosis through targeting the mitogen-associate protein kinase 4 (MAP4K4) pathway. MiR-30d-5p is abundantly expressed in cardiomyocytes, with its levels significantly increasing in response to various stressors. Furthermore, it is highly concentrated in extracellular vesicles (EVs) released by cardiomyocytes, suggesting a role in intercellular communication. In studies using αMHC-MerCreMer-RosaTmG reporter mice, it has been demonstrated that miR-30d-5p can transfer from cardiomyocytes to fibroblasts, facilitating the crosstalk between these two cell types and thereby contributing to cardiac remodeling. Mechanistically, miR-30d-5p released from cardiomyocytes in EVs inhibited fibroblast proliferation and activation by directly targeting integrin α5, and thereby mediating paracrine signaling to cardiac fibroblasts [48]. 

MiR-181c-5p, an miRNA that has been linked to cardiovascular complications [49,50,51], was shown to be significantly increased in the blood of patients with DM and HFpEF. MiR-181c has been shown to target and decrease the expression of Parkin RBR 3 ubiquitin protein ligase (PRKN) and SMAD7, thereby enhancing the pro-fibrotic response and contributing to adverse cardiac remodeling [52]. Additionally, the overexpression of miR-181c has been linked to increased reactive oxygen species (ROS) production and elevated mitochondrial calcium levels, both of which are associated with oxidative stress leading to HF. In experimental models, mice deficient in miR-181c/d exhibited reduced cardiac hypertrophy compared to wild-type controls when subjected to high-fat diet (HFD) [53]. 

The expression of miR-24 was shown to be increased in humans with obesity [47] and in murine cardiac tissue with pressure overload-induced hypertrophy. The overexpression of miR-24 resulted in the direct targeting and decreased expression of p27, a cyclin-dependent kinase inhibitor (CDKI) that regulates cell cycle, in neonatal rat ventricular myocytes (NRVM) in vitro and in cardiac tissue in vivo. This interaction promoted cell cycle progression into the S phase, thereby contributing to the development of cardiac hypertrophy by promoting myocyte growth [54]. Whether the dual regulation of miR-24 by obesity and pressure-overload induced hypertrophy exacerbate the progression of cardiac remodeling into overt HF remains to be studied.

Although several studies to date have demonstrated that miRNAs regulate cardiac remodeling by targeting genes involved in cardiac hypertrophy and fibrosis, miRNA profiling in the context of obesity and HF could provide valuable insights into the molecular mechanisms driving disease progression. Understanding how miRNA expression changes in human plasma and cardiac tissue in response to obesity, and the mechanistic impact on cardiac remodeling during the progression into HF, may inform the development of targeted therapeutic approaches.

## 3. Angiogenesis

Coronary angiogenesis plays a crucial role in maintaining cardiac vascularization and perfusion in response to pathological and physiological conditions. Impaired coronary angiogenesis, particularly reduced capillarization, is strongly associated with pathological cardiac hypertrophy and ventricular dysfunction. 

Microvascular dysfunction and increased metabolic demand in obese patients not only lead to inadequate myocardial perfusion and hypoxia [55] but also exacerbates cardiac dysfunction in pressure overload-induced cardiac hypertrophy, accelerating the onset of HF. Conversely, pro-angiogenic stimulation has been shown to delay HF progression, highlighting its importance in preserving cardiac function [56,57,58,59]. 

Endothelial cells (ECs) located at the inner most layer of all blood and lymphatic vessels [60], are key mediators of angiogenesis through their regulation of EC proliferation and migration. Obesity-induced endothelial dysfunction is driven by several factors, including dysregulated endothelial nitric oxide (NO) production, a vasodilator that modulates EC survival, proliferation, and migration [61,62,63,64]. Additionally, increased endoplasmic reticulum (ER) stress [65], elevated circulating free fatty acid levels [66,67,68], and the activation of pro-inflammatory pathways [69,70,71,72] exacerbate endothelial stress, ultimately impairing vascular function. 

MicroRNAs have been identified as key regulators of EC function in obesity. However, their role in modulating angiogenesis in HF within the context of obesity remains poorly understood. Notably, miR-221-3p expression has been negatively correlated with cardiac function in patients with HF while in vivo neutralization of miR-221-3p promoted cardiac angiogenesis in a murine model of TAC-induced HF through the targeted regulation of hypoxia-inducible factor-1 α (HIF-1α) [73]. Additionally, miR-221-3p expression correlated positively with BMI and waist circumference, while negatively associating with insulin sensitivity. Its expression was reduced in the plasma of pediatric patients following weight loss [74], implicating that the obesity-associated regulation of miR-221-3p may play an important role in the modulation of angiogenesis in HF. 

Genetic deletion of miR-216a-5p, a pro-angiogenic microRNA upregulated in obese women [75], has been linked to impaired cardiac microvascular EC function. In a TAC model of chronic pressure overload-induced HF, miR-216a-5p deletion resulted in capillary rarefaction, reduced myocardial oxygenation, and accelerated HF onset. This effect was mediated through the dysregulation of EC-angiogenic functions and direct targeting of phosphatase and tensin homolog (PTEN) and beclin-1 (BECN1). Although the role of PTEN in ECs remains insufficiently characterized, knockout of miR-216a-5p in mice exhibited reduced PTEN expression and decreased capillary density. Furthermore, in vitro neutralization of miR-216a-5p led to increased BECN1 levels and enhanced autophagy. Together, these findings demonstrate that miR-216a-5p is essential for maintaining angiogenesis, particularly under conditions of pressure overload and myocardial infarction-induced HF. With studies showing its upregulation in obese humans [76], miR-216-5p may be an important positive regulator of EC angiogenic functions in the myocardium under obesity. 

MiR-34a was shown to mediate capillary rarefaction by targeting the HIF-1α/VEGF signaling axis in a murine model of right ventricular failure (RVF) and was negatively correlated with capillary density in patients with RVF [77]. The expression of miR-34a was significantly increased in patients with impaired glucose tolerance, prediabetes, and metabolic syndrome [78], suggesting the possible involvement of miR-34a in the etiology of obesity-associated angiogenic dysfunction in HF. 

MiR-665-3p levels are elevated in cardiac ECs of patients with HF. The inhibition of miR-665-3p or the overexpression of its target, CD34, in a mouse model of TAC improved EC proliferation, angiogenesis, and cardiac function. Furthermore, TAC upregulates the transcription factor Sp1, which has been shown to regulate miR-665-3p expression in human umbilical vein endothelial cells (HUVECs) [79]. Notably, miR-665-3p expression was significantly increased in the liver and hepatic cells of mice with DIO [80]. However, its role in HF in the context of obesity needs to validated in prospective studies in obese patient cohorts with HFpEF or HFrEF.

Finally, elevated miR-204 levels in ECs contribute to endothelial dysfunction by suppressing SIRT1 expression, leading to increased endothelial endoplasmic reticulum (ER) stress, reduced Cav1 (caveolin-1) levels, and impaired vasorelaxation [81]. Moreover, HFD increased vascular miR-204 expression while decreasing Cav1. Given Cav1’s protective role in shielding cardiac ECs from hemodynamic stress, these findings suggest a potential anti-angiogenic role of miR-204 in heart disease and obesity [82]. However, whether Cav1 is a direct target of miR-204 remains unclear. Interestingly, miR-204 exhibits a contrasting role under cardiac stress. In a mouse model of TAC-induced HF, miR-204 was found to be upregulated in response to cardiac stress, where it facilitated cardioprotection by modulating Apelin signaling and preventing hypertrophy [83]. 

These findings highlight the dual nature of angiogenic miRNAs in HF: some contribute to endothelial dysfunction while others offer protective effects under stress conditions. Precision-based therapies that selectively target these miRNAs could help restore vascular homeostasis and delay HF progression, particularly in patients with obesity-related cardiovascular risk. Further research into the context-dependent roles of these miRNAs will be essential for developing targeted interventions that enhance LV angiogenesis. 

## 4. Apoptosis

In end-stage HF, loss of cardiomyocytes occurs alongside fibrosis and cellular hypertrophy. Apoptosis, a programmed form of cell death, is a key mechanism contributing to cardiomyocyte loss, with its rate increasing as HF progresses. In contrast, myocyte size, volume, and sarcomere number expand in pathologic hypertrophy. The rate of apoptosis typically rises with age and is exacerbated by pressure overload, gradually leading to HF as cumulative myocyte loss impairs cardiac function [84]. Obesity-associated oxidative stress, elevated levels of pro-inflammatory cytokines, such as TNF alpha, norepinephrine, and angiotensin II [85], along with dysregulated leptin signaling [86,87], further promote cardiomyocyte apoptosis. Obesity also activates FAS and mitochondria-dependent apoptosis, potentially accelerating of HF progression [88,89].

Several miRNAs that were dysregulated in obesity were also shown to modulate cardiac apoptotic pathways in murine models of pressure overload-induced hypertrophy. For example, miR-150-5p knockout mice subjected to myocardial infarction (MI) developed worsened cardiac remodeling and increased apoptosis, ultimately leading to HF. MiR-150-5p directly targets the pro-apoptotic factor small proline-rich protein 1A (SPRR1A), inhibiting its activity. Heart tissue from patients with HFrEF has shown increased SPRR1A expression [90], while it was also upregulated in murine myocardium post-TAC [91]. In support of these findings, the overexpression of miR-150-5p in murine heart conferred protection against cardiac hypertrophy and fibrosis following TAC [92]. While in vivo studies have demonstrated the cardioprotective role of miR-150-5p, its expression was significantly downregulated in patients with HF, serving as a predictive biomarker correlated with established HF parameters, such as N-terminal pro-B-type natriuretic peptide (NT-proBNP) [93,94]. Interestingly, the expression of miR-150-5p was also downregulated in the serum of obese individuals compared to those with normal BMI [95,96], while an opposing trend was observed in the pediatric population with increased miR-150-5p expression in the circulation of obese children with high hepatic fat and serum triglyceride [95,97], which could be due to differences in lipid metabolic regulation and inflammatory responses in children and adolescents, highlighting the complexity of miRNA regulation. Whether overexpression of miR-150-5p can mitigate apoptosis in the setting of obesity and help with the progression of HF remains to be determined. 

Similarly, miRNA-222-3p expression was increased in a murine model of pressure overload-induced hypertrophy, where cardiac-specific overexpression following TAC improved cardiac function. Transcriptomic profiling revealed that miR-222-3p targets the p53-up-regulated modulator of apoptosis (PUMA), a pro-apoptotic gene [98]. Notably, PUMA-deficient mice that underwent TAC maintained cardiac function similar to the sham group lasting up to four weeks [99]. In Dahl salt-sensitive (DS) obese rats, treatment with the SGLT2 inhibitor ipragliflozin effectively reduced cardiac hypertrophy and concurrently suppressed miR-222-3p expression [46]. Additionally, miR-222-3p expression was found to be lower in myocardial biopsies from patients with aortic stenosis and dilated cardiomyopathy (DCM), indicating its dysregulation in pressure overload and hypertrophic conditions [100]. Interestingly, miR-222-3p expression was elevated in morbidly obese individuals [101], and its increased expression persisted even after weight loss [102]. This suggests a potential link between obesity, cardiac stress, and apoptotic regulation, which may play a role in the adaptive response to pressure overload-induced hypertrophy.

Conversely, miR-125b-5p expression was downregulated in the plasma of obese humans [101] and in murine cardiac tissue subjected to TAC. Increased miR-125b-5p expression has been shown to suppress apoptosis by reducing the levels of anti-apoptotic genes, such as caspase-3 and Bax, while elevating the pro-apoptotic factor BCL2 through targeting Bcl-2 homologous antagonist/killer 1 (BAK1) [103]. These findings highlight a critical interplay between obesity and apoptosis in cardiac pathology, where dysregulated microRNA expression may contribute to HF. Together, these results underscore the potential of microRNAs as biomarkers and therapeutic targets in obesity-associated cardiac dysfunction, particularly in the context of pressure overload-induced hypertrophy and HF.

## 5. Inflammation

Inflammation plays a crucial role in the development and progression of HF through various pathways, including the activation of pro-inflammatory cytokines and adhesion molecules, which trigger the migration of macrophages, neutrophils, monocytes, and lymphocytes. This process is accompanied by impaired microvascular dilation, fibroblast, myofibroblast proliferation, and capillary rarefaction [104], thereby contributing to the systolic and diastolic dysfunction in HF, especially in the setting of HFpEF [105]. 

Several strategies have been explored to reduce inflammation in chronic HF, focusing on targeting TNF-α due to its pivotal role in HF pathogenesis [106,107,108]. However, clinical trials of TNF-α receptor antagonists in HF have reported excess mortality, suggesting that TNF-α receptor antagonism has negative cardiovascular effects. Postulated reasons for these setbacks include the bidirectional role of inflammatory cytokines in HF pathogenesis, potential adverse effects of high doses, and limited impact on myocardial inflammation. Therefore, targeting inflammatory markers in HF remains challenging, and current strategies to regulate cytokine imbalance may not be optimal.

Understanding the contribution of miRNAs to inflammation in HF, especially in the setting of obesity, may offer more etiology-specific therapeutic candidates. The expression of miR-181b was significantly increased in the white adipose tissue (WAT) of obese mice and humans [109], while its expression was decreased in the serum and heart tissue of obese mice with cardiac hypertrophy and LV dysfunction [110]. Additionally, miR-181b levels inversely correlated with the levels of high-sensitivity C-reactive protein, a key inflammatory marker [111], further highlighting its potential role in modulating inflammation-related cardiac dysfunction. In a rat model of HF, miR-181b expression was downregulated in both the early and advanced stages of the disease. Early HF was associated with cardiac hypertrophy and reduced miR-181b expression, while advanced HF exhibited increased levels of pro-inflammatory cytokines (TNF-α, IL-1β, and IL-6), which further reduced miR-181b expression in the cardiac tissue of rats treated with isoproterenol. In vitro studies demonstrated that miR-181b overexpression could inhibit LPS-induced inflammatory responses in neonatal cardiomyocytes, indicating a regulatory role for miR-181b in inflammation in HF [111]. In a rat model of sepsis, the overexpression of miR-181b reduced inflammation and cardiomyocyte apoptosis by targeting high-mobility group box-1 protein (HMGB1) and thereby reducing cardiac injury [112]. Importantly, its expression is increased with methotrexate (MTX), an immunosuppressive drug, decreasing the endothelial inflammation in obese mice [113]. 

MiR-155 also plays a crucial role in obesity and HF pathogenesis by regulating inflammatory responses. Studies have indicated that the expression of miR-155 was strongly upregulated in human and mouse models of myocarditis, and its expression was primarily detected in infiltrating macrophages and T lymphocytes. The inhibition of miR-155 reduced the cardiac infiltration of monocyte-derived macrophages, the activation of T lymphocytes, and myocardial damage, while improving cardiac function by targeting an inhibitor of dendritic cell antigen presentation to T cells [114]. Additionally, miR-155 expression was increased in the subcutaneous adipose tissue of obese individuals compared to those with normal BMI and positively correlated with TNF-α expression, possibly by targeting PPAR-γ [115]. On the other hand, research has also explored the role of miR-155 in the obesity paradox, which suggests that individuals with a higher BMI may have better outcomes in cardiovascular disease despite obesity being a known risk factor for heart disease. ApoE knockout (KO) mice, a standard model of atherosclerosis, exhibited increased miR-155 expression in the mice aorta after 3 weeks on HFD and 6 hours of lipopolysaccharide (LPS) stimulation. This upregulation promoted atherogenesis by inducing EC activation and enhancing pro-inflammatory macrophage responses. Furthermore, an ApoE/miR-155 double KO mouse model provided significant insights into the complex interplay among obesity, atherosclerosis, and non-alcoholic fatty liver disease (NAFLD), where, despite increased adipogenesis, these mice showed a reduction in atherosclerotic lesions [116]. Concurrently, they developed NAFLD and enhanced adipogenesis. These observations underscore the pivotal role of miR-155 in modulating inflammation and lipid metabolism, thereby influencing the pathogenesis of both cardiovascular and metabolic diseases. Further investigations are warranted to elucidate the underlying mechanisms of this paradox, particularly focusing on the interconnections between inflammatory pathways and obesity-related cardiovascular conditions. 

MiR-223-3p has been extensively studied in HF and is recognized for its multifaceted roles in disease progression. In HF, the expression patterns of miR-223-3p vary depending on the stage of the disease. In individuals with early-stage HF, particularly those with concurrent diabetes, miR-223-3p expression was downregulated in the heart [117]. In contrast, patients with end-stage HF showed significantly elevated levels of miR-223-3p expression, suggesting that this increase may be associated with advanced stages of HF [118]. An upstream regulator of miR-223-3p, the circular RNA known as circSnap47, plays a role in HF progression by targeting the miR-223/MAPK signaling axis [119]. Other upstream regulators of miR-223-3p, including the transcription factors PU.1 and C/EBPs [120], may even work synergistically to enhance inflammation, as they are targeted by the NFκB signaling axis [121,122]. This regulatory influence of circSNAP47 on miR-223-3p suggests it could be a critical factor in the worsening of HF. In obesity, miR-223-3p exhibits differential expression in various types of adipose tissue. In one study, miR-223-3p was significantly upregulated in human visceral adipose tissue [123], often associated with obesity-related inflammation. Conversely, miR-223-5p was markedly reduced in subcutaneous adipose tissue following weight loss from gastric bypass surgery [124]. While it is not fully understood if miR-223-3p directly impacts HF in response to the low-grade inflammation commonly seen with obesity, its ability to target a key component in inflammatory processes, namely FBXW7, a subunit of the ubiquitin protein ligase complex called SKP1-cullin-F-box and a well-known suppressor of Toll-like receptor 4 (TLR4) signaling in macrophages [123,125], indicated that miR-223-3p might help modulate the inflammation in HF in the context of obesity. 

In a murine HF model achieved by coronary artery ligation, adenoviral overexpression of miRNA-130a improved LV ejection fraction by reducing TNF-α expression in the myocardium while also decreasing the expression of endothelin-1 (ET-1) in plasma [126]. Furthermore, the profiling of umbilical cord serum miRNAs associated with obesity identified hepatic exosome-derived miR-130a-3p as a regulator of energy metabolism in adipose tissue, where its expression was increased in the circulation of children with an increased risk for obesity [127].

Finally, the inhibition of miR-146a was shown to improve cardiac function, enhance hemodynamics, and reduce heart remodeling in myocardial infarction (MI)-induced HF [128]. It regulates cholesterol metabolism and helps control chronic inflammation in response to an atherogenic diet by limiting proinflammatory signaling in endothelial and bone marrow-derived cells [129]. MiR-146a-deficient mice fed HFD exhibited increased weight gain, adiposity, and hepatic steatosis compared to the wild-type control, suggesting a protective role of miR-146a against DIO [129]. 

The interplay among miR-155, miR-130a, miR-181b, miR-146a, and miR-223-3p underscores the importance of miRNAs in the regulation of inflammation in both obesity and HF. For example, miR-181b appears to have anti-inflammatory properties, and its upregulation could help mitigate HF progression. Meanwhile, miR-155, which is associated with obesity-related inflammation, might serve as a potential biomarker or target for interventions aimed at reducing cardiovascular risk in obese individuals. Moreover, such interplay also underscores the complexity of HF pathogenesis, necessitating a cautious approach in developing miRNA-based therapies. Given that some miRNAs, such as miR-155, may have paradoxical effects, the modulation of these pathways may yield unintended consequences due to their pleiotropic effects. Developing effective, etiology-specific therapies will require a deeper understanding of microRNA signaling dynamics to ensure targeted interventions that mitigate inflammation without compromising essential immune or metabolic functions. 

## 6. Lipotoxicity

Obesity-associated excessive lipid accumulation in non-adipose cells of the cardiovascular system can lead to cell death, a phenomenon known as lipotoxicity [130]. In the heart, lipotoxicity impairs the heart’s metabolic flexibility, exacerbating oxidative stress and inflammation, ultimately increasing the risk of HF [131,132,133]. Among the key regulators of lipotoxicity, several miRNAs have emerged as critical post-transcriptional modulators of lipid metabolism and cellular stress responses in the heart. 

One such miRNA, miR-494-3p, was downregulated in the myocardium of mice with DIO. Notably, the overexpression of miR-494-3p reduced triglyceride uptake, oxidative stress, and apoptosis by modulating the JunD/PPARγ axis in neonatal rat ventricular myocytes (NRVMs) exposed to palmitate. Mechanistically, Ago2 immunoprecipitation and 3'- UTR luciferase assays in NRVMs confirmed JunD as a direct target of miR-494-3p. Interestingly, JunD expression was significantly elevated in the myocardium of obese patients, correlating with reduced miR-494-3p levels, increased myocardial triglyceride accumulation, upregulated PPARγ expression, and left ventricular dysfunction [134] suggesting that miR-494-3p plays a protective role against obesity-induced lipotoxicity by regulating key metabolic pathways. 

Similarly, elevated miR-451 levels were detected in neonatal rat cardiac myocytes (NRCMs) treated with palmitate, as well as in the heart tissues of DIO mice. Furthermore, neutralizing miR-451 in NRCMs improved palmitate-induced lipotoxicity. Supporting these in vitro findings, the cardiac-specific deletion of miR-451 alleviated HFD-induced cardiac hypertrophy and improved contractile function by targeting calcium-binding protein 39 (CAB39), a scaffold protein of liver kinase B1 (LKB1) and an upstream kinase of AMP-activated protein kinase (AMPK). This regulation was also associated with a reduction in mammalian target of rapamycin (mTOR) expression [135], suggesting miR-451’s potential involvement in metabolic stress responses.

While miR-451 appears to exacerbate lipotoxicity, miR-184 expression has been shown to be elevated in the plasma of patients with obesity [136], with its expression significantly reduced in the myocardium of mice fed a HFD for 24 weeks or in vitro in H9C2 cardiomyoblasts treated with palmitic acid. Mechanistically, miR-184 targeted and reduced the expression of phosphatidic acid phosphatase (PLPP3), which decreased diacylglycerol (DAG), a precursor for triglyceride biosynthesis, and increased insulin sensitivity. This was associated with increased glucose consumption, enhanced AKT phosphorylation, and improved insulin signaling in cardiomyocytes [137]. Whether these effects are tissue-specific, and how they may be leveraged for therapeutic interventions in obesity-associated cardiac dysfunction remains to be elucidated. 

In summary, obesity-related metabolic cardiomyopathy and HF primarily arise from the accumulation of lipotoxic by-products within cardiac cells, leading to myocyte apoptosis and impaired contractility. Lipotoxicity, driven by an excessive buildup of triglycerides, free fatty acids, and other lipid intermediates, causes significant cardiac damage by generating reactive oxygen species (ROS) and activating apoptotic pathways. Studies have suggested that mitigating cardiac lipotoxicity may help alleviate obesity-associated heart dysfunction. 

Understanding the mechanisms by which microRNAs influence lipid biosynthesis, lipid metabolism, and the accumulation of these harmful products in the heart is critical. Despite growing interest in the role of microRNAs in lipotoxicity, it is still unclear which metabolic pathways they most strongly regulate, which lipid products are the most harmful, and how lipotoxicity interacts with inflammation and glucose metabolism. In addition, miRNAs have emerged as potential prognostic biomarkers of lipotoxicity and lipid metabolism, particularly in ischemic heart disease and HF in the obese population. This is because changes in lipid metabolism and cellular fuel usage often precede more recognizable alterations, such as cardiac remodeling and functional impairments, which are typically detected through imaging and other diagnostic methods.

## 7. Mitochondrial Dysfunction

Maintaining mitochondrial homeostasis for endogenous energy production is essential for proper cardiac function. The dysregulation of miRNAs and their involvement in mitochondrial dysfunction has garnered significant attention, especially in the context of HF and cardiomyopathies. This highlights the critical role of mitochondrial function in cardiac health [138]. Furthermore, the association between obesity, as a metabolic disorder, and mitochondrial dysfunction has been extensively investigated. Research has particularly emphasized how mitochondrial dysfunction impairs various organs in the context of obesity [139]. However, a gap remains in understanding the specific role of miRNAs linking mitochondrial dysfunction to obesity-related cardiac dysfunction. 

One such miRNA, miR-195, has been strongly implicated in mitochondrial dysfunction and plays a role in obesity and HF by targeting mitochondrial health and metabolic function. miR-195 expression is significantly increased in a murine TAC model and in human HF [140,141,142]. Elevated miR-195 levels are associated with the hyperacetylation of mitochondrial enzymes, resulting from its downregulation and direct targeting of sirtuin 3 (SIRT3), a mitochondrial deacetylase. SIRT3 helps protect mitochondrial enzymes from acetylation, preserving their activity and stability. By directly targeting SIRT3, miR-195 increased protein acetylation, leading to diminished mitochondrial enzymatic activity, impaired energy production, and exacerbated mitochondrial dysfunction in HF [142]. Beyond its role in HF, elevated miR-195 levels have also been linked to metabolic syndrome, where its expression was correlated with increased BMI [7,143] and hypertension [7,144]. These findings suggest that miR-195 may serve as a potential biomarker for metabolic and mitochondrial health. 

MiR-194 expression was significantly elevated in the circulation of age-matched patients with a BMI greater than 28 compared to lean individuals with a BMI below 23. This correlated with higher levels of N-terminal pro-brain natriuretic peptide (NT-proBNP) and reduced ejection fraction (EF), both of which are key indicators of LV dysfunction [145]. To explore the functional impact of miR-194, a mouse model of HFD-induced obesity was used, where the systemic neutralization of miR-194 using a viral sponge enhanced mitochondrial activity. This resulted in increased ATP production and basal oxygen consumption of cardiac tissue, ultimately resulting in better EF and reduced cardiac hypertrophy [145]. Additionally, in an isoproterenol-induced cardiac hypertrophy model, calcineurin A (CnA) was identified as a direct target of miR-194 in cardiac myocytes [146] further supporting miR-194’s role in mitochondrial and cardiac dysfunction.

An exosomal microRNA, miR-122, previously identified as being secreted by the liver, has been linked to obesity-related cardiomyopathy. Wang and colleagues demonstrated that miR-122 expression was elevated in the plasma samples of patients with obesity, showing a significant positive correlation with cardiac dysfunction. Mechanistically, miR-122 directly targets the mitochondrial protein ARL-2 (ADP-ribosylation factor-like 2), a regulatory GTPase. The dysregulation of this interaction leads to mitochondrial impairment and abnormal cardiac remodeling, as evidenced by in vitro studies on primary cardiomyocytes and in vivo mouse models with DIO [147]. 

MiR-181c is a microRNA classified as a MitomiR (mitochondrial microRNA), a term that describes microRNAs translocated to the mitochondria upon maturation in the cytoplasm. Elevated levels of miR-181c have been observed in heart samples from patients with cardiac dysfunction, and its presence was also increased in the mitochondrial fraction of cardiac tissue obtained from mice with DIO, where it was associated with myocardial lipidosis. In addition to its role in regulating lipidosis in the myocardium, miR-181c can alter lipid metabolism and biosynthesis in the liver under DIO conditions [144]. In a whole-body genetic knockout mouse model of miR-181c/d, cardioprotective effects were demonstrated in DIO by reducing ROS production and limiting mitochondrial calcium accumulation. Mechanistically, miR-181c directly binds to the 3′-UTR of mt-COX1, indirectly downregulating the mitochondrial Sp1/MICU1 axis that controls calcium influx [53,144,148].

In human plasma samples with obesity, increased miR-29a expression was correlated with cardiac dysfunction. The treatment of primary cardiomyocytes with circulating human exosomal miR-29a induced mitochondrial dysfunction, while systemic neutralization of miR-29a restored mitochondrial function, improved ATP production, and reduced IκB phosphorylation in cardiac tissue, thus improving the cardiac function of mice with DIO [149]. Additionally, miR-29 genetic deficiency in a double KO mice model (miR-29a/b1^−/−^) was associated with the development of HFpEF. In these miR-29a-deficient mice, the authors reported systemic hypertension, a metabolic shift from fatty acid utilization to glucose utilization, along with mitochondrial abnormalities in cardiac tissue [150]. These effects are likely mediated through previously reported direct targets of miR-29a, PGC1α [151], a crucial regulator of mitochondrial biogenesis and function. 

Excessive caloric intake, increased mitochondrial substrate load, or mitochondrial dysfunction that impairs the effective dissipation of the proton gradient can lead to elevated ROS production. This results in cellular damage, mtDNA mutations, and apoptosis. Perturbations in cardiac energy metabolism represent some of the earliest changes observed in the myocardium during the progression of metabolic syndrome-related heart dysfunction. Despite these insights, the identification of miRNAs regulated by these early metabolic disturbances remains underexplored. A deeper understanding of these miRNAs could significantly enhance their diagnostic utility in heart disease, especially as early biomarkers of metabolic disruptions. However, significant gaps persist in our understanding of miRNAs and their connection to obesity-related HF. In particular, the functional roles of mitochondria-associated miRNAs and their interactions with nuclear-encoded miRNAs require further investigation to elucidate their contributions to obesity-related cardiac dysfunction. Emerging evidence indicates that miRNAs may regulate critical processes, such as mitochondrial fusion–fission dynamics, mitochondrial metabolism, calcium homeostasis, and other pathways involved in mitochondrial dysfunction. Nevertheless, many of these mechanisms, including the regulation of mitophagy and mitochondrial dynamics by miRNAs, remain poorly characterized. There is a pressing need for well-designed preclinical and clinical studies that leverage advanced methodologies capable of isolating mitochondrial genetic material with minimal contamination. Such approaches could provide crucial insights into miRNA-mediated changes in the mitochondrial genetic landscape and its functional implications. Addressing these gaps will be crucial for advancing our understanding of how obesity-induced mitochondrial dysfunction contributes to cardiac disease and for identifying novel therapeutic and diagnostic opportunities that target mitochondrial miRNAs.

## 8. Conclusions and Perspectives

MicroRNAs are present in various biological fluids and exhibit remarkable stability, making them easily extractable from biological samples, such as blood [152]. They are differentially regulated both during the progression of many disease states and in response to various HF therapies and can simultaneously target multiple genes and pathways (Table 1), providing a potentially valuable non-invasive means for accurate diagnosis, prognosis, treatment guidance, and treatment response evaluation [153]. Despite challenges in targeted delivery to tissues and specific cell types, advances in RNA chemistry and delivery technologies, particularly nanoparticle systems, have facilitated the transition of microRNA-based approaches into clinical settings [154]. 

With a growing understanding of the roles various microRNAs play in diseases, engineered microRNA therapeutics hold promise for revolutionizing therapeutic strategies by targeting multiple proteins and enzymes involved in disease progression, rather than targeting them individually [156,157,158,159].

Achieving targeted delivery to specific cell types or tissues is crucial for the effective development of miRNA-based therapeutics. One approach involves conjugating miRNA modulators with peptides or antibodies to facilitate their homing to particular cell types [160]. Alternatively, lipid-based formulations can encapsulate antimiRs or miRNA mimics, enhancing their cell specificity [161]. In the absence of highly specific delivery methods, device-based approaches, like stents or catheters, local injections, and ectopic delivery, can help overcome some delivery obstacles. Given the established role of cardiac catheterization in clinical practice, such approaches could be highly feasible in HF. The currently reported delivery techniques for antisense oligonucleotides already include inhalation for lung delivery, enema formulations for gut delivery, intraventricular or intrathecal administration for brain delivery, and direct intraocular delivery for ocular conditions. In addition to conventional protocols, additional methods for administering miRNA-based drugs are being investigated. An encouraging strategy involves incorporating miRNA therapeutics into biodegradable 3D matrices, which can be surgically implanted into affected tissues [162]. Further advancements are anticipated, as 3D matrices are also tailored for delivering various nucleic acid-based therapies and conventional drugs through different routes, such as edible or injectable carriers [163].

MiRNAs hold potential as diagnostic or prognostic tools due to their ability to reflect origin, and other pathological factors through their expression profiles. They serve as precise molecular markers owing to their stability and resistance to RNase degradation, likely due to their small size. There is a growing availability of miRNA markers for diagnosing various cardiovascular disease states. For example, in murine models of autoimmune and viral myocarditis, circulating mmu-miR-721 has been shown to be significantly elevated. Plasma levels of its human homolog hsa-miR-Chr8:96 successfully distinguished myocarditis from myocardial infarction and from healthy individuals across various cohorts [164]. MiR-19b-3p has emerged as a promising prognostic biomarker of acute HF. Elevated levels of circulating miR-19b-3p at the onset of HF have been associated with poorer outcomes, including increased mortality and higher rates of hospital readmission. Its expression was correlated with markers of LV hypertrophy and fibrosis, indicating its potential role in cardiac remodeling processes [165]. Such ongoing research highlights the potential for microRNAs to serve as valuable biomarkers and offer additional diagnostic or prognostic benefits beyond the standard markers, which are sensitive but lack specificity for HF, such as brain natriuretic peptide (BNP) and N-terminal pro-BNP (NT-proBNP). This is particularly useful for the non-invasive diagnostic detection of HF in patients with DM and obesity [164,166].

AntimiR therapies have demonstrated promising results after the positive outcomes observed in a few Phase I and Phase II clinical trials. The miR-29 family (consisting of miR-29a/b/c) is known to reduce expression levels in fibrotic conditions, where it acts to inhibit the accumulation of extracellular matrix [167]. MRG-110, a mixer of LNA and DNA antagomiR molecules, modified with phosphorothioate internucleotide bridges, targets miR-92, aiming to address cardiovascular diseases, such as HF. By inhibiting miR-92a, MRG-110 was designed to stimulate angiogenesis as a potential treatment approach in a Phase I trial. In a clinical Phase 1b study, cardiac miR-132-3p levels were shown to increase in patients with HF, while CDR132L, an antisense oligonucleotide specific to miR-132 attenuated HF in preclinical models. The study, conducted on a small group of patients, demonstrated high efficacy, significant decreases in cardiac fibrosis, and improved cardiac function, providing encouraging preclinical observations in humans [168]. Increased levels of low-density lipoprotein cholesterol (LDL-C) are a major risk factor for obesity-induced HF. Recently, FDA-approved siRNA-based therapy inclisiran showed efficacy in reducing circulating LDL-C by targeting PCSK9. PCSK9 binds to LDL receptors, inducing conformational changes that inhibit receptor recycling, thereby reducing LDL receptors on the cell surface. This results in elevated LDL-C levels in the blood, contributing to hypercholesterolemia. Mechanistically, inclisiran, which is designed with triantennary GalNAc, specifically targets asialoglycoprotein receptors (ASGPRs) on hepatocytes, facilitating its uptake into liver cells, where it degrades PCSK9 mRNA [169].

The effectiveness of miRNA therapeutics depends on the route of administration and successful intracellular delivery to avoid systemic side effects beyond the targeted cells and tissues. One notable example of this was a synthetic miR-34a mimic that led to severe immune-related side effects and was terminated from clinical trials [155]. Despite obstacles to integrating therapeutic miRNAs into clinical practice, these challenges are identifiable. Novel approaches that target miRNAs during the development of HF may pave the way for preventative strategies and targeted therapies.

## Figures and Tables

**Table 1:** Key microRNAs (miRNAs) implicated in heart failure (HF), their targets, and mechanisms of action. This table summarizes miRNAs that play significant roles in HF and highlights their validated or potential utility as therapeutic targets. Each miRNA is associated with specific molecular targets and contributes to HF progression through distinct pathophysiological mechanisms, including cardiac hypertrophy, fibrosis, angiogenesis, apoptosis, and inflammation. * = Potential target, # = targeted by approved pharmacological agents.

miRNAs	Targets	Functions	References	Models
		**Cardiac Hypertrophy and Fibrosis**		
miR-410-5p	SMAD7	Promotes cardiac fibrosis	[43]	Rat
miR-141-3p	PSEN1	Promotes cardiac hypertrophy and fibrosis	[44,45]	Rat, H9C2
miR-144-3p #	----	Protects against cardiac hypertrophy and fibrosis #: Downregulation by ipragliflozin	[44,46]	Rat
miR-30d	ITGA5	Inhibits cardiac fibroblast proliferation and activation	[36,47,48]	Human, rat, mouse, H9c2 cells
miR-181c	PRKN/ SMAD7 *	Increases (ROS) and mitochondrial calcium influx	[49,50,51,52,53]	Human, mouse, H9C2 cells, NMVMs
miR-24	CDKN1B	Increases cardiac hypertrophy	[47,54]	Human, rat, NRCMs
miR-181c	MT-CO1	Promotes ROS and mitochondrial calcium influx, protective against fibrosis in vivo	[53,144,148]	Human, mouse, rat
miR-150	SPRR1A	Protects against cardiac hypertrophy and fibrosis	[90,91,92,93,94,95,96,97]	Human, mouse, H9c2, HL-1
miR-184	PLPP3	Increases insulin sensitivity under obesity and LV mass	[136,137]	human, mouse
miR-451	CAB39	Exacerbates HFD-induced cardiac hypertrophy	[135]	Human, mouse, NRCMs
miR-122	ARL2	Promotes mitochondrial impairment in cardiomyocytes and cardiac hypertrophy	[147]	Human, mouse, primary mouse CMs
miR-195	SIRT3	Promotes mitochondrial dysfunction and hypertrophy in a TAC model and obesity	[13,140,141,142,143,144]	Human, mouse, rat
miR-194	CNA1	Neutralization increased ATP production, basal oxygen consumption, and reduced hypertrophy	[146,147]	Human, mouse, primary mouse CMs, H9c2
		**Angiogenesis**		
miR-221	HIF1A	Inhibits cardiac angiogenesis	[73,74]	Human, mouse, HUVECs
miR-216a	PTEN/ BECN1	Promotes EC proliferation/angiogenesis under HF and PO	[75,76]	Human, mouse, HUVECs
miR-34a	----	Promotes angiogenic dysfunction in obesity and HF	[77,78,155]	Human, mouse, primary mouse cells, HMVEC
miR-665	CD34	Inhibits EC proliferation under HF	[79,80]	Human, mouse, HCMEC,HUVEC
miR-204	SIRT1/ CAV1 *	Promotes endothelial dysfunction under HFD, can be protective against hypertrophy in a TAC model	[81,82,83]	Human, mouse, NRCMs, H9C2
		**Apoptosis**		
miR-222 #	PUMA	Protective against PO-induced cardiac hypertrophy/HF #: Ipragliflozin downregulates	[46,98,99,100,101,102]	Human, mouse, NRCFs
miR-125b	BAK1	Pro-apoptotic in a TAC model	[101,103]	Human, mouse, primary mouse cells
miR-494-3p	JUND/ PPARG *	Decreases oxidative stress, triglyceride uptake, and apoptosis	[134]	Human, mouse, NRVMs
		**Inflammation**		
miR-181b #	HMGB1	Regulates inflammatory response in HF #: Activated by methotrexate (MTX)	[109,110,111,112,113]	Human, rat, mouse, primary rat cells
miR-155	PPARG	Regulates inflammatory response in obesity and HF	[40,114,115,116]	Human, mouse, primary mouse cells
miR-223	FBXW7	Modulates inflammation in HF under obesity	[117,118,119,120,121,122,123,124,125]	Human, rat, H9C2
miR-130a	----	Regulates energy metabolism in adipose tissue, reduces TNF-α expression in murine HF	[126]	Rat
miR-146a	----	Regulates cholesterol metabolism and limits pro-inflammatory signaling	[127,128]	Human, rat
miR-29a	PPARGC1A	Promotes mitochondrial dysfunction and inflammation	[149,150,151]	Human, mouse

## Data Availability

No new data were created or analyzed in this study. Data sharing is not applicable to this article.

## Abbreviations

AMPK	AMP-activated protein kinase
ARL-2	ADP-ribosylation factor-like 2
ASGPRs	Asialoglycoprotein receptors
BECN1	Beclin-1
BNP	Brain natriuretic peptide
CAB39	Calcium-binding protein 39
CDKI	Cyclin-dependent kinase inhibitor
Cav1	Caveolin-1
DAG	Diacylglycerol
FAS	Fas cell surface death receptor
GTPase	Guanosine triphosphatase
HCMEC	Human cardiac microvascular endothelial cells
HMGB1	High-mobility group box-1 protein
HIF-1α	Hypoxia-inducible factor-1 α
LDL-C	Low-density lipoprotein cholesterol
LKB1	Liver kinase B1
LPS	Lipopolysaccharide
MAP4K4	Mitogen-activated protein kinase 4
mTOR	Mammalian target of rapamycin
NO	Nitric oxide
NT-proBNP	N-terminal pro-brain natriuretic peptide
NMVM	Neonatal mouse ventricular myocyte
NRVM	Neonatal rat ventricular myocytes
NRCM	Neonatal rat cardiomyocytes
NRCF	Neonatal rat cardiac fibroblasts
PUMA	p53-upregulated modulator of apoptosis
PRKN	Parkin RBR 3 ubiquitin protein ligase
PTEN	Phosphatase and tensin homolog
PLPP3	Phosphatidic acid phosphatase
PSEN1	Presenilin 1
SGLT2	Sodium-glucose cotransporter 2
SIRT3	Sirtuin 3
SPRR1A	Small proline-rich protein 1A
TLR4	Toll-like receptor 4
